# Upgrading of Gleason score on radical prostatectomy specimen compared to the pre-operative needle core biopsy: An Indian experience

**DOI:** 10.4103/0970-1591.60445

**Published:** 2010

**Authors:** Rishi Nayyar, Prabhjot Singh, Narmada P. Gupta, Ashok K. Hemal, Prem N. Dogra, Amlesh Seth, Rajeev Kumar

**Affiliations:** Department of Urology, All India Institute of Medical Sciences, Ansari Nagar, New Delhi - 110 029, India

**Keywords:** Cancer prostate, gleason grade, gleason score, needle core biopsy, radical prostatectomy

## Abstract

**Objectives:**

To assess the accuracy of Gleason grading/scoring on preoperative needle core biopsy (NCB) compared to the radical prostatectomy (RP) specimen.

**Materials and Methods:**

Data of NCB and RP specimens was analyzed in 193 cases. Gleason grade/scoring was done on both NCB and RP specimens. Sixteen cases were excluded for various reasons. The Gleason scores of the two sets of matched specimens were compared and also correlated with the PSA, age, and number of needle biopsy cores. The overall change was also correlated with the initial score on NCB.

**Results:**

The mean age and PSA were 63.3±2(5.27) years and 18.48±2(28.42) ng/ml, respectively. The average Gleason score increased from 5.51 ± 2(1.52) to 6.2 ± 2(1.42) (*P*<0.02). The primary grade increased in 57 (32.2%) cases. Overall, 97 (54.8%) cases had an increase in Gleason score. Five other cases had a change from 3 + 4 = 7 to 4 + 3 = 7. Change in Gleason score was significantly more if the score on NCB was ≤6 or number of needle cores was ≤6. Besides, 28 cases had perineural invasion, 16 had capsular invasion (pT3_a_), and 4 had vascular invasion on RP specimen.

**Conclusions:**

There is a significant upgrading of Gleason score on RP specimens when compared with NCB. This trend may be correlated positively with lower initial Gleason score on preoperative biopsy and the lower number of cores taken.

## INTRODUCTION

The Gleason score of prostate adenocarcinomas is not only an important preoperative predictor of cancer behavior but it is also used to help guide treatment.[1] It is not uncommon for the final Gleason grade/ score of the radical prostatectomy (RP) specimen to differ from the one seen in preoperative needle core biopsy (NCB). It is important to know the inaccuracies of the needle biopsy Gleason scoring, since it helps in better prognostication and choice of treatment.[1] These inaccuracies must be kept in mind before putting the patient on a watchful waiting protocol, otherwise these patients may run the risk of inadequate treatment at a relatively higher TNM stage. It is also important for better evaluation of studies that compare results of surgical treatment with those of radiation or conservative management.[1,2] From an outcome research point of view, it is important to recognize that a stratification of patients by Gleason score on postoperative specimen may prove correct in patients undergoing RP, while in patients undergoing radiation or conservative management some of the well-differentiated cancers diagnosed on NCB could actually be moderately and poorly differentiated, and some of the moderately differentiated might be poorly differentiated, thus favoring RP in a direct comparison of long-term treatment efficacy. This is also true in a reverse sense when NCB results in overgrading. We conducted a study in our institution to assess these inaccuracies, specifically to assess for the upgrading of Gleason grade/score on RP specimens and if there are some indicators to predict such upgrading.

## MATERIALS AND METHODS

Data were collected for 193 cases that underwent RP after detection of localized prostate cancer in our institution during the study period from July 2002 to December 2008. The RP was done by open retropubic, laparoscopic (transperitoneal or extraperitoneal), or robotic technique –34, 32, and 127 cases, respectively. Thirteen cases that had already (preoperatively) received some form of hormonal treatment (leutinizing hormone releasing hormone agonists or androgen receptor blockers) were excluded from the purview of this study because such therapy may interfere with the correct interpretation of the tumor and its grade in the postoperative specimen. Three cases had rare variants of cancer prostate— adenocarcinoma of the large prostatic ducts in two and papillary cystadenocarcinoma in one. These cases were also excluded from the final analysis. The remaining 177 patients constituted the study group. In these patients, Gleason scoring was done on the NCB specimens and then on the final RP specimens. Presence of other known prognostic parameters like presence of the perineural or vascular invasion and capsular invasion was also noted. The record of number of cores taken for NCB was also kept. In cases where only one of the cores was positive for tumor, we calculated the score on NCB by doubling the grade of the cancerous site. In one of the cases, there was only one focus of residual tumor in the RP specimen, which was again assigned a Gleason score by doubling its grade. The Gleason scores of the two sets of matched specimens were then compared and also correlated with the preoperative PSA levels, number of preoperative biopsy cores, and age. Statistical analysis was done using appropriate statistical tests (One way ANOVA, student's *t* test) with the help of SPSS version 13.

All Gleason grading and scoring was done by senior uropathologists at our institution. For few patients who had already undergone biopsy outside, the slides were again reviewed thoroughly by our pathologists. The average duration between transrectal biopsy and RP was about 9 weeks, ranging from 6 to 14 weeks.

## RESULTS

Total 177 cases of the study group underwent RP for clinically localized carcinoma prostate. They had a mean age of 63.3 years [±2(5.27)] (range 45–76 years) and PSA of 18.48 ng/ml [±2(28.42)] (range 0.3–68.3 ng/ml). The mean NCB Gleason score was 5.51 [±2(1.52)] (range 2–9). An upgrading of Gleason scoring was seen on RP specimens from the same patients such that the mean Gleason score became 6.2 [±2(1.42)] (range 2–9). Thus an average upgrading of the Gleason score by 0.69 was noted. This difference was significant at a *P* < 0.05.

When only the primary grade was considered, 57 cases out of 177 (32.2%) showed an upgradation in their primary score and 19 (10.7%) showed downgradation. Both the up-or downgradation was upto a maximum of two units, average upmigration being 1.35 and average downmigration being 1.14. Considering only the secondary grade, upgrading and downgrading was seen in 48 (27.1%) and 8 (4.5%) cases, respectively. Average upmigration was 1.17 (range 1–2). Downmigration was by 1 unit in all 8 cases with a decrease in secondary grade.

Besides, the final histopathology report showed perineural invasion in 28 cases (all of which had a final Gleason score of ≤ 7), capsular invasion (pT3_a,b_) in 16 cases, and vascular invasion (tumor emboli in peritumoral tissue) in 4 cases. Out of these, the NCB could pick up only five cases of perineural invasion. Although all 28 cases of perineural invasion on RP specimen had a final Gleason score of ≥7, only 17 of these had Gleason score of ≥7 on the NCB. Out of other 11 patients with NCB Gleason score of ≤6,8 had Gleason score of 6 reported on NCB. Seven out of these 11 cases were diagnosed on ≤6 biopsy cores. Average number of NCB in these cases was 6.3 (range 2–12, median 6) and average number of positive cores was 2 (range 1–3, median 2).

Overall 97 (54.8%) patients had an increase in their Gleason score, the increase ranging from 1 to 4 units. Their average pre-and postoperative Gleason score was 4.79 [±2(1.18)] and 6.35 [±2(1.21)], respectively. Such patients had an average age of 62.79 years [±2(11.47)] and average PSA of 18.3 ng/ml [±2(22.97)] as against 63.3 years [±2(5.27)] and 18.48 ng/ml [±2(28.42)] for whole study population. This difference in age and PSA was not clinically significant at a *P* value of 0.05.

Nineteen (10.7%) patients had a decrease in their Gleason score on the final RP histopathology compared to the preoperative biopsy score. The decrease in these patients ranged from 1 to 3 units.

Sixty-one (34.4%) patients had the same Gleason score as on the NCB. Out of these, five patients had a change of Gleason grading from 3 + 4 = 7 to 4 + 3 = 7. The reverse change, that is, 4 + 3 = 7 to 3 + 4 = 7, was seen in two cases. Overall, eight cases had an increase and five had a decrease in their primary grade with total score remaining the same in NCB and RP specimen. This increase or decrease in primary grade was to a maximum of 1 unit.

The number of cases with the Gleason score ≤6 in the NCB group was 126, significantly (*P* > 0.05) more than the 102 seen in the RP group. In this group of patients who had an initially lower (≤6) Gleason score on NCB, the average increase in the score was 0.92 units. In the remaining 51 cases (initial Gleason score ≥7), 5 cases had an increase in Gleason score by 1 unit and 5 also had a decrease by 1 unit. Thus, the overall average change in Gleason score in such patients was zero. Therefore, it can be said that the initial low Gleason score was associated with a subsequent significant rise in the final scoring on postoperative histopathology, while an initial high Gleason score on preoperative biopsy was not associated with further rise in final scoring on RP specimen. By further dividing the study population into three groups based on preoperative Gleason score, viz., less than 6, equal to 6, and more than 6, it was found that the average increase in Gleason score was 0.76, 0.95, and 0, respectively. This suggests that the increase is significantly (*P* > 0.05) more likely to occur in the group with preoperative score of 6. This fact holds importance in the management planning for cases where NCB Gleason score is reported as 6.

Total 104 patients out of 177 patients were diagnosed on 6 or less needle cores, while the rest 73 were diagnosed on 7–13 extended core biopsies. The mean change in the Gleason score on the postoperative biopsy was 1.07 [±2(0.96)] and 0.87 [±2(0.85)] for the patients with ≤6 and ≥7 core biopsies, respectively. This difference in the average Gleason score between the two groups is again statistically significant at *P* < 0.05. This suggests that increasing the number of cores reduces the chances of upgrading on the subsequent RP specimen. In other words, extended core biopsy, rather than the sextant biopsy, better correlates with the subsequent scoring on the RP specimens.

When patients were subgrouped according to their PSA levels [Table 1], 54 had PSA value of more than 20 ng/ml. Average change in Gleason score in patients with serum PSA 20 ng/ml was 0.73 [±2(0.70)]. On the other hand, average change in Gleason score in patients with serum PSA >20 ng/ml was 0.64 [±2(0.62)]. This difference in upgrading of Gleason score in the two groups with PSA <20 and >20 was not significant at a *P* value of 0.05.

**Table 1:** Subgrouping of study cases (*N = *177) of radical prostatectomy according to preoperative serum PSA level

PSA (ng/ml)	No. of cases

<4	13
4.1-10	63
10.1-20	47
>20	54

## DISCUSSION

Gleason grading has been the most popular system for pathological grading of carcinoma of the prostate.[3] It is based on the glandular architectural pattern (grade 1–5). Gleason score gives the sum of grades of two most prevalent architectural patterns of the tumor (score 2–10). Gleason score has become an important criterion to predict tumor behavior and guiding treatment. However, Gleason scoring on NCB is frequently lower than the actual scoring on RP specimens. This fact has an important bearing on treatment decisions particularly if the patient is being considered for the watchful management protocol. It is also important to better understand studies involving direct comparison of RP with radical radiotherapy or watchful management because the results may be biased by the presence of some relatively higher grade disease patients in the nonsurgical arm. The undergrading of the tumor on NCB is related to the limited tissue sampling on trucut biopsies, not hitting the representative area on trucut biopsy, or pathologist's bias/ inhibition to label high-grade disease.[1,2,4 ] Moreover, it is not always possible to predict perineural/capsular/vascular invasion on NCB.

In our study, we found a significant difference (*P*<0.02) between the average Gleason score on the NCB (5.51) and that on RP specimen (6.2). As much as 97 out of 177 (54.8%) patients had shown an increase in Gleason score. Our results compare well with other series in the literature.[1,5–9] This upmigration was demonstrable not only in terms of increase in total Gleason score but also as an increase in primary Gleason grade with or without the increase in the total Gleason score; and also as a demonstration of perineural, perivascular, or pericapsular invasion. Although all 28 cases of perineural invasion on RP specimen had a final Gleason score of ≥7, only 17 of these had Gleason score of ≥7 on the NCB. Thus we might have failed to identify those 11 patients with Gleason score of <7, which had perineural invasion as a poor prognostic marker, if they were to be kept under watchful management based only on their Gleason score on NCB. Eight out of these 11 cases had a preoperative NCB Gleason score of 6, which brings to light the importance of considering early RP in patients with NCB Gleason score of 6.

The upgrading in our study was not related to the patient's age or PSA value. However the initial lower score on preoperative biopsy and also the lower number of cores of preoperative biopsy were related to it, and such cases had a relatively higher degree of upmigration. It has been proved in previous studies that larger the number of cores, better is the correlation between the Gleason grading on NCB and RP.[1,6,10–12] This fact signifies the importance of taking extended core/saturation biopsy before selecting any particular patient for the watchful waiting protocol for the management of carcinoma prostate. Also the chance of upgrading is significantly more if the score on NCB is 6 than with the score of <6. Such an observation has also been done earlier by Pinthus *et al*.[13] Therefore careful selection is definitely warranted for these cases with Gleason score of 6 before putting them on watchful waiting protocol. This fact also holds importance in the definition of insignificant prostate cancer. To overcome these limitations arising from the Gleason grading/scoring on NCB, tumor volume has also been added to the original definition of ‘insignificant prostate cancer’.[14] Our finding of upgrading as well as the fact that some microscopic poor prognostic factors like capsular or perineural infiltration may be missed on NCB present a case for considering early prostatectomy in patients who are otherwise considered candidates for watchful management but are also on the borderline for radical treatment.

The decrease in the Gleason grading and scoring in some patients might be explained with the occurrence of crush artifacts on the NCB. The grading is primarily based on the structural pattern of the tissue and it would be understandably difficult to interpret in the presence of crush artifacts. Moreover, the grade in the NCB might not be the grade from the site with the highest tumor volume in the RP specimen. Thus, the higher grade on NCB might actually be representative of the secondary or tertiary grade but not the primary grade. In our study, all the Gleason scoring on the NCB was done by finding out the highest Gleason score from all the cores for that particular patient. In literature, both the highest Gleason score and the Gleason score from the site with the highest tumor volume have been said to be equally and significantly predictive of final Gleason score on the subsequent RP specimen.[15] Lastly, the overgrading on NCB could also be the result of the pathologist's reading error.

Basically, NCB sample cannot be representative of the whole prostate gland and would thus frequently differ from the final score on the RP specimen. We have found a significant upgrading in a large number of patients with downgrading in only a few. Based on our study data, we would recommend an extended core/saturation biopsy before embarking on the watchful waiting protocol and discuss the possible undergrading on NCB with the patient to help him better understand the prognosis and make decisions regarding further treatment. Such undergrading is more likely in patients with a lower reported grade and lesser number of cores on needle biopsy. A limitation of our study is that not all histopathological examinations were conducted by a single pathologist, which could induce an error of difference of reporting by different individuals. However, all reporting was done by the senior consultants with good experience in reporting prostate samples. Moreover, pre-and postoperative reporting for each case was done by the same pathologist for that case, which would tend to minimize the effect of such an error.

## CONCLUSION

There is a significant upgrading of Gleason scoring on RP specimens when compared with NCB. This trend does not correlate with patient's age or PSA value. The sextant biopsy increasingly tends to undergrade the tumor than the extended core/saturation biopsy. Upgrading is maximum in cases with NCB Gleason score of 6. Perineural or microscopic pericapsular invasion is frequently missed on NCB.
